# Vulvar and Perianal Condyloma Superimposed Inflammatory Linear Verrucous Epidermal Nevus: A Case Report and Review of the Literature

**DOI:** 10.1155/2013/261574

**Published:** 2013-12-17

**Authors:** Sümeyra Nergız Avcioğlu, Sündüz Özlem Altinkaya, Mert Küçük, Hasan Yüksel, Selda Demircan-Sezer, Gonca Uçar

**Affiliations:** Department of Gynecology and Obstetrics, School of Medicine, Adnan Menderes University, 09100 Aydın, Turkey

## Abstract

Inflammatory linear verrucous epidermal nevus (ILVEN) is a benign cutaneous hamartoma characterized by intensely erythematous, pruritic, and inflammatory papules that occur as linear bands along the lines of Blaschko. There is a considerable clinical and histological resemblance between ILVEN and linear psoriasis, lichen striatus, linear lichen planus, and invasion of epidermal nevus by psoriasis. The pathogenesis of ILVEN is unknown. It is regarded as a genetic dyskeratotic disease reflecting genetic mosaicism. Here, a case of vulvar and perianal condyloma superimposed ILVEN is presented.

## 1. Introduction

Inflammatory linear verrucous epidermal nevus (ILVEN) is a relatively uncommon benign cutaneous hamartoma described by Altman and Mehregan in 1971 [1]. It is characterized by intensely pruritic, erythematous, inflammatory papules coalescing into well-demarcated verrucous plaques in a linear distribution. Patients seek help for its symptoms and cosmetic appearance [2]. Medical management is extremely variable but unfortunately does not result in complete recovery. On the other hand, surgical excision of lesions is not preferable due to extensive scarring and relapse of disease [3]. Here,a case of vulvar and perianal condyloma superimposed ILVEN is presented.

## 2. Case Presentation 

**Figure 1:**
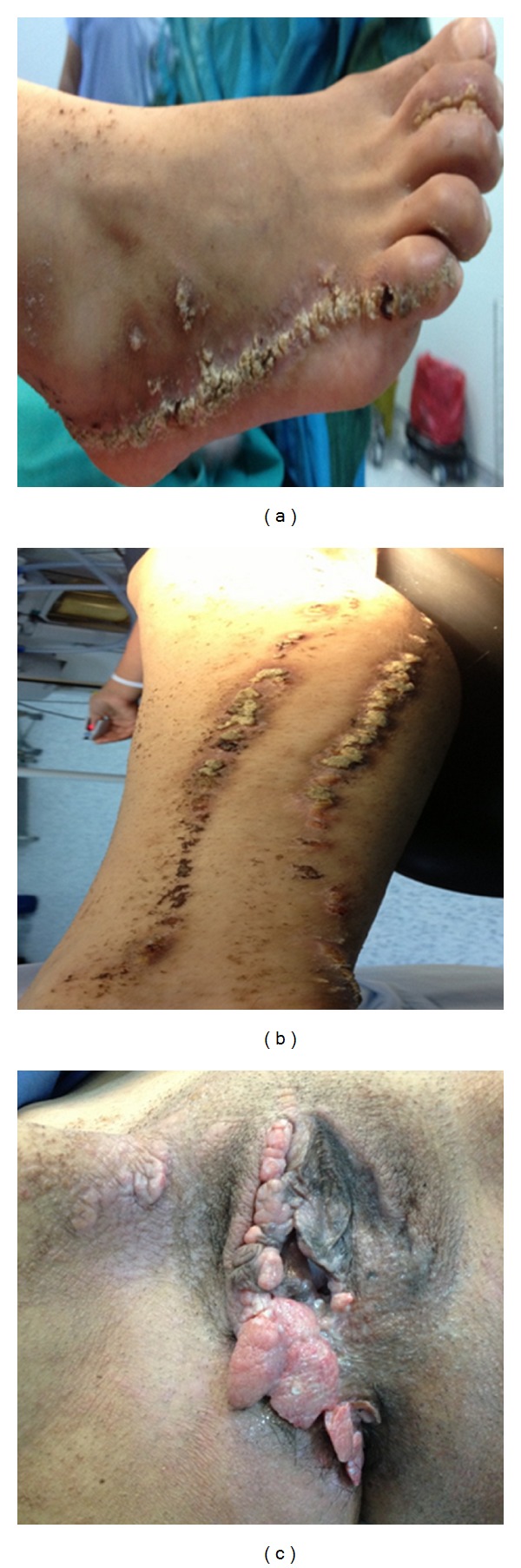
Vulvar and perianal condyloma superimposed ILVEN seen in the right side of the body.

A 21-year-old virgo patient, has presented with a huge amount of vulvar and perianal condylomas. Surgical excision was planned. But in physical examination of patient, erythematous scaly plaques were seen unilaterally on the right side of body. Detailed medical history revealed that, erythematous lesions, of linear or grouped distribution, involving only right side of body; upper trunk, axilla, and lower extremity unilaterally had existed since a few years after birth. Lesions were extremely pruriginous and aggravated with heat. There were no pathological antecedents or previous family history of the disease. Dermatological examination revealed papular lesions and erythematous plaques with areas of scaling and crusts, linearly distributed at the right side of the body. (Figures 1(a), 1(b), and 1(c)). Disease was diagnosed as ILVEN by histopathological examination. Besides, laboratory tests including C3, C4, immunoglobulin (Ig)A, IGM, IGE, IGG anti-HAV IGM, and anti-HAV IGG were performed. Laboratory investigations showed mild anemia (hemoglobin 10.5 g/dL), an elevated erythrocyte sedimentation rate (45 mm/h), and an elevated C-reactive protein level (36.3 mg/L, normal < 8.0 mg/L). Liver enzymes, renal function test results, and immunoglobulin levels were normal. Results of tests for antinuclear antibodies and rheumatoid factor were negative. Also vulvar and perianal condylomas were examined for human papilloma virus (HPV) serotypes by HPV DNA polymerase chain reaction method. HPV 11 was detected. Surgical excision was performed for vulvar and perianal condylomas and pathology result confirmed the diagnosis. For treatment of ILVEN, lesions in the present case were resistant to topical steroid therapy and CO_2_ therapy and surgical excision was recommended.

## 3. Discussion

Inflammatory linear verrucous epidermal nevus (ILVEN) is a rare form of epidermal nevus. The cause and pathogenesis are unknown [2]. ILVEN is more common in females and may be familial [4–6]. Classic criteria for the diagnosis of ILVEN as established by Altman and Mehregan [1] in 1971, and later modified by Morag and Metzker [7] in 1985: (1) unilateral, linear verrucous eruption accompanied by intense pruritus, (2) early age of onset, and (3) refractoriness to treatment. Atypical presentations have been described in the literature, including widespread, bilateral distribution and relatively late onset in adulthood [8]. Isolated reports also reveal a familial occurrence, as demonstrated by the development of ILVEN in a mother and her daughter [4]. In the present case report, typical presentation, early onset, and unilateral appearance of disease were observed.

In a 1983 review, Fox and Lapins [9] compared the efficacy of various methods of treatment as reported in the literature and used by the authors. ILVEN was found to be resistant to topical steroids (either with or without occlusion), tretinoin cream, 5-fluorouracil (5-FU) cream, podophyllin ointment, and tar preparations. So, success with medical management is extremely variable and inconsistent. Furthermore, any improvement tends to be only temporary unless maintenance therapy on a regular basis is continued. In the literature, there have been also researches about success of CO_2_ therapy in treatment of ILVEN [10]. Similar to this, lesions in the present case were resistant to topical steroid therapy and CO_2_ therapy and surgical excision was recommended.

The interesting issue in the present case report is the occurrence of huge amount of vulvar and perianal condylomas in sexually inactive, virgo patient with ILVEN. Several experiences induced us to consider genital HPV infection as an expression of a local immunodeficiency [11]. Cell mediated immunity has a role in HPV infection [12]. A number of studies have investigated the ability of cytokines, particularly TGF-b, TNF, the interferons alpha, beta, gamma, and IL1 to inhibit the proliferation in vitro of both normal and HPV-transformed keratinocytes, as well as inhibiting expression of HPV genes including the early genes E6 and E7 [13]. Recently, the role of helper T lymphocytes in providing protection against the development of HPV-associated lesions by measuring T-cell proliferative responses [14] or IL-2 release [15] has been investigated. Empirical evidence for the importance of cell-mediated immunity in control of HPV infection also comes from an extensive body of the literature documenting the increased prevalence of HPV infection and associated diseases among immunosuppressed populations, including those with iatrogenic immunosuppression such as renal transplant recipients and individuals with human immunodeficiency virus (HIV) infection. Most studies have suggested that advanced disease and greater immunological deficit are associated with higher prevalence and persistence of HPV infection [16]. Correlated with the mentioned literature, in the present case, disease of ILVEN was suspected to occur due to a malfunction in the immune system of the patient. A group of tests about immunity of the patient was performed but results were inconclusive. However, molecular investigations about cell mediated immunity of the patient have been continued.

On the other hand, the pathogenesis of ILVEN is unknown. It is regarded as a genetic dyskeratotic disease reflecting genetic mosaicism. Because of its clinical and histological similarity to linear psoriasis, shared pathogenic traits such as the central involvement of T cells may be hypothesized [17]. In the literature, ILVEN was determined as a clinical variant of verrucous epidermal nevus and associated with dysregulation of interleukin 1, interleukin 6, tumor necrosis factor, and intercellular adhesion molecule 1 [18]. Besides, elevated ICAM-1, ELAM-1, and HLA-DR expression in ILVEN suggested an inability to downregulate the inflammatory infiltrate [19]. Favoring the mentioned literature, ILVEN has been described previously in association with other disorders, including autoimmune thyroiditis [20] lichen amyloidosis [21], HIV1 infection [22], and skeletal defects [23] although this last association is in dispute [24]. Besides, two children with the combination of ILVEN and arthritis were reported [25]. In the present case, coexistence of ILVEN and HPV infection establishes clues about immunological basis of ILVEN. However, of course, recent studies and case reports about etiopathogenesis are preliminary and not conclusive. In conclusion, molecular researches to investigate immunological basis of ILVEN, including high number of cases are needed.
